# Ustekinumab for steroid-refractory pancolitis in a biologically naive child: A case report and literature review

**DOI:** 10.1097/MD.0000000000033061

**Published:** 2023-03-03

**Authors:** Marouf Alhalabi

**Affiliations:** a Syrian Board in Gastroenterology, a Gastroenterologist at the Gastroenterology Department of Damascus Hospital, Damascus, Syria.

**Keywords:** child, Crohn disease, inflammatory bowel disease, pediatric, ulcerative colitis, Ustekinumab

## Abstract

Ustekinumab is not recommended for the treatment of children with inflammatory bowel disease, but its off-label use is increasing despite a lack of pediatric pharmacokinetic data. The purpose of this review is to evaluate the therapeutic effects of Ustekinumab on children with inflammatory bowel disease and to recommend the best treatment regimen. Ustekinumab was the first biological treatment for a 10-year-old Syrian boy with steroid-refractory pancolitis who weighed 34 kg. A 260 mg/kg (~6 mg/kg) intravenous dose was followed by 90 mg of subcutaneous Ustekinumab at week 8 (induction). The patient was supposed to receive the first maintenance dose after twelve weeks, but after ten weeks, he developed acute severe ulcerative colitis which was managed according to treatment guidelines, except receiving 90 mg of subcutaneous Ustekinumab when he was discharged. The maintenance dose of 90 mg subcutaneous Ustekinumab was intensified to every 8 weeks. Throughout the treatment period, he achieved and maintained clinical remission. In pediatric inflammatory bowel disease, a dose of intravenous ~6 mg/kg of Ustekinumab is a common induction regimen, while children weighing < 40 kg may require a dose of 9 mg/kg. For maintenance, children may require 90 mg of subcutaneous Ustekinumab every 8 weeks. The outcome of this case report is interesting with improved clinical remission and highlighting the expansion of clinical trials on Ustekinumab for children.

## 1. Introduction

In children, ulcerative colitis (UC) is a chronic and progressive disease characterized by extensive colonic involvement.^[1]^ Aside from the potentially harmful effects of chronic corticosteroids, complications such as colon cancer may arise during the disease long period of coexistence.^[2]^ When used in conjunction with mesalamine or sulfasalazine, curcumin may be a safe and effective therapy for maintaining remission in UC. More studies are needed to identify the right dose, delivery method, formation, and intervention time.^[3–5]^ Conventional medical treatments frequently fail to control pediatric UC, whereas the only approved biological treatments in children are infliximab and adalimumab, both of which are necrosis factor antagonistsα (anti-TNFα).^[6]^ A significant proportion of children respond initially, then lose response or develop intolerant to anti-TNFα.^[6]^ As a result, there is a significant unmet need for pediatric inflammatory bowel disease (IBD)for novel treatment. Ustekinumab is an interleukin-12/23 inhibitor that is approved for the treatment of moderate to severe IBD in adults.^[7–10]^ Despite the lack of pediatric pharmacokinetic data, the off-label use of Ustekinumab in the treatment of IBD children has continued to be reported. The failure of earlier previous biological experiments was used as justification. As with adults, the induction regimen was weight-based dosing of 6 mg/kg followed by 90 mg of subcutaneous (SC) Ustekinumab after 8 weeks, with 90 mg of SC Ustekinumab administered every 8 or 12 weeks for maintenance.^[7,8]^ We report the outcome of a case of steroid-refractory pancolitis, in a 10-year-old boy, who was treated with as the first biological treatment with Ustekinumab as the first biological treatment. We used the same induction regimen and a 12 weeks maintenance regimen.

## 2. Case presentation

A 10-year-old Syrian boy with steroid-refractory pancolitis was evaluated after presenting with mild abdominal pain and bloody, nocturnal diarrhea (6–8 times), The estimated (pediatric ulcerative colitis activity index [PUCAI] = 55 points). The diagnosis was confirmed by an ileocolonoscopy and colon biopsies with histology 5 months ago.^[6]^ His weight was 34 kg and stands 140 cm tall (body mass index = 17.35kg/m^2^), he had no family history of IBD and no surgical history. His past medications included mesalamine at 60 to 80 mg/kg/day, and prednisone 1 mg/kg once daily, up to 40 mg.^[6]^ Screening for infections, which is recommended before prescribing biological therapies, came back negative.^[6,11]^ Ustekinumab was used as the first and only available biological treatment with intravenous 6 mg/kg (260 mg) and 90 mg of SC Ustekinumab after 8 weeks (induction). He achieved clinical remission (PUCAI = 10) points after 8 weeks, and 90 mg of subcutaneous Ustekinumab was administered, with an appointment for the next dose after 12 weeks.^[7,12]^ Ten weeks after the induction, (26 weeks from starting), he presented with tachycardia, abdominal pain, and 6 episodes of nocturnal and bloody diarrhea. The estimated PUCAI was 75 points.^[13]^ We verified oral mesalamine compliance by counting both consumed and leftover pills, child tests are shown in Table 1. Stool cultures, Clostridium difficile toxin testing, E. coli, Cryptosporidium, and microscopy for ova and parasites were all negative. A Sigmoidoscopy disclosed spontaneous bleeding as well as multiple ulcers. Colon biopsies were negative for Clostridium difficile and Cytomegalovirus infection. The estimated Mayo score was 11 points. He was managed according to treatment guidelines,^[14]^ besides receiving 90 mg of SC Ustekinumab when he was discharged, and the 90 mg of SC Ustekinumab maintenance period was escalated to every 8 weeks. The assignment for week 52 revealed clinical remission (PUCAI = 5 points), a partial Mayo score of 0 points, and fecal calprotectin of 20 ug/g. He gains a few kilograms, his weight became 43 kg and his BMI was 22kg/m^2^. Other tests were viewed in the Table 1.

**Table 1:** Child laboratory test results.

Laboratory test	Reference range	W 0	W 26	W 52
WBC, mm^3^	4500–10500	12800	13600	6000
Hemoglobin, mg/dL	13–18	9.8	10.4	13.8
Platelets, mm^3^	(150–450) × 10^3^	452	461	152
Serum creatinine,mg/dL	0.5–1.3	0.6	0.6	0.5
CRP, mg/L	0–5	102	92	2
Urea, mg/dL	10–45	31	29	21
Glucose, mg/dL	6–115	72	80	78
Na, mmol/L	134–148	139	137	138
K, mmol/L	3.5–5	4.1	4.2	3.9
ALT, U/L	5–40	16	14	18
AST, U/L	5–40	18	13	17
Fecal calprotectin, µg/g	<150	380	451	20
ESR, mm/hr	≤20	103	109	11

CRP = C-Reactive Protein, ESR = erythrocyte sedimentation rate, Na = Sodium, WBC = white blood cell.

## 3. Discussion

In adults with IBD who responded to induction therapy, Ustekinumab demonstrated efficacy and safety.^[7–10,15]^ It was also linked to a high rate of drug persistence as well as improvements in clinical and biochemical measures in adults who had previously failed other biologics.^[16,17]^ Children with IBD, particularly those with active disease, require early biologic therapy; however, a significant proportion of children respond initially, and then lose response or become intolerant to biological therapy.^[6]^ When compared to adult patients, the availability of new treatments is frequently delayed. Clinical trials in children are more difficult to conduct than in adults, simply duplicating an adult randomized controlled trial in children is ineffective.^[18]^ Although adalimumab is effective in the treatment of pediatric UC, the findings came from a retrospective cohort study, resulting in a weak recommendation for pediatric UC patients who have not previously received infliximab.^[6]^ The only evidence for golimumab in pediatric UC was an open-label pharmacokinetic study and 2 case reports, resulting in a weak recommendation.^[19–22]^ Except in a few instances, Ustekinumab was administered after other biologics had failed.^[23–25]^ Infliximab was the most commonly reported failed biologic, as expected, and Table 2 summarizes reports on Ustekinumab in pediatric IBD patients. Beginning in 2016, and prior to the approval of intravenous induction, only SC induction without SC maintenance was used.^[26,27]^ Then, following SC induction, use SC maintenance.^[28,29]^ For the first time in children, Two large cohorts used an intravenous induction dose of 6 mg/kg, similar to the adult,^[7,8]^ followed by an 8-week SC maintenance dose for the first time in children. One of these cohorts was directed limited to children with infliximab refractory UC,^[30]^ whereas CD and a few UC children were included in the other cohort.^[23]^ UniStar a phase 1 clinical trial that evaluated the pharmacokinetics, safety, and efficacy of Ustekinumab in CD children reported its result in the middle of 2021. Its findings supported a 6 mg/kg induction dose of Ustekinumab for children weighing more than 40 kg, but recommended a 9mg/kg induction for children weighing < 40 kg.^[25]^ UniStar was not designed to determine Ustekinumab maintenance dose for children, but its findings are consistent with other pediatric biologics pharmacokinetic studies that recommended higher induction dose in low-weight children.^[19,20,31]^ As the clearance of Ustekinumab varies with body weight, history of anti-TNFα failure, and the presence of drug antibodies, and is unrelated to the nature of the disease.^[32]^ Adult patients who responded to induction are given a maintenance dose of 90 mg of SC Ustekinumab every 12 or 8 weeks.,^[7,8]^ and it appeared that the every-8-week interval appeared had a numerically higher percentage of clinical remission.^[9,10,15]^ UNIFI and UNITI, on the other hand, were not designed to assess differences between maintenance regimen.^[7–10]^ In nearly all cases, the off-label use of Ustekinumab in pediatric IBD patients was associated with a history of biological treatment failure. The need to intensify the maintenance dose of the SC Ustekinumab was linked to the persistence of active colonic disease, and the inability to achieve biochemical response.^[24,33]^In our case, however, the cause was a loss of response. Because we live in a resource-constrained country, we cannot measure Ustekinumab levels. We assumed that because we ruled out disease mimickers and the child had responded to induction, so it assumed that was due to low Ustekinumab concentrations. Thus, intensification is effective among loss of responders,^[34–36]^ and the majority of children responded to Ustekinumab escalation.^[37]^ In our case, he achieved clinical and biomarker remission by adhering to the standard of care following the the results of STARDUST trial. STARDUST adds to our understanding of the role of Treat to Target (T2T) in CD treatment, and is expected to apply to UC patients.^[38]^ More research is needed to understand how to manage patients who are in clinical response or remission but have persistent evidence of inflammation.^[39]^ However, when compared to other biologics, Ustekinumab was safe with no noticeable side effects, adverse events, or activation of opportunistic diseases.^[9,10,23,25,30]^ It should be noted that after the start of Ustekinumab, a case of disseminated tuberculosis was activated. Ustekinumab was started without a new chest X-ray or a repeat interferon gamma release assays test because the child was switched from another biologic.^[40]^ Also, another case described drug-induced lung disease after starting Ustekinumab; the child complained of shortness of breath, chest pain, and cough.^[41]^ Adult clinical trials are expanding the therapeutic options for IBD patients, but the time lag between adult and pediatric drug approval results in significant off-label use due to a lack of knowledge about optimal pediatric dosing regimens. Clinical trials in bio-naive and bio-expert IBD children particularly those who are young and low-weight are needed. The long-term persistence of remission and the ease of the regimen without a concomitant immunomodulator, suggest that Ustekinumab will be a valuable addition to the therapeutic options for pediatric IBD. An induction regimen of 6 mg/kg or 9 mg/kg. for children weighing < 40 kg, then followed by 90 mg of SC Ustekinumab every 8 weeks which can be intensified to every 6 or 4 weeks. The outcome of this case report is interesting with improved clinical remission and highlighting the expansion of clinical trials on Ustekinumab for children.

**Table 2:** Reports summarize Ustekinumab in pediatric IBD patients.

Authors	Report type	IBD	Prior biologic	UST induction	UST maintenance
Rinawi et al^[26]^	Case report	CD	IFX, ADA	3 SC doses of Ustekinumab (22.5 mg–1.3 mg/kg) at mo (0, 1, 3)	None
Cameron et al^[27]^	Case report	CD	IFX, ADA	180 mg of SC UST and then 90 mg of SC UST at w 2.	None
Bishop et al^[28]^	Case series	CD	IFX, ADA	90 mg of SC UST at w 0 and 4	90 mg of SC UST q8w
M. Chavannes et al^[29]^	Cohort	CD	IFX, ADA, CIMZIA, GOL, or VDZ	Six different regimens of SC UST were used	90 mg of SC UST q8w
Dayan et al^[23]^	Cohort	CD: N = 42, UC: N = 4, IBDU: N = 6	Only 10 patients were biologic-naive	Intravenous weight based induction 6 mg/kg	90 mg of SC UST q8w
Dhaliwal et al^[30]^	Cohort	UC	IFX, and ADA or VDZ	Intravenous weight based induction 6 mg/kg	90 mg of SC UST q8w, or 45 mg q8w for smallest child
Rodríguez-Mauriz et al^[33]^	Case report	CD	ADA	Intravenous induction, 360 mg of UST	90 of SC UST q8 then q6w
Y. Fujita et al^[24]^	Case report	CD	None	Intravenous induction,260 mg of UST	90 mg of SC UST q12w then q8w
Kim et al^[42]^	Cohort	CD	At least 1 of IFX, ADA, CIMZIA, or VDZ	Intravenous weight based induction 6 mg/kg	90 mg of SC UST every 8 some patients SC UST q6w or q4w
Rosh et al^[25]^	Phase 1, double-blind, study	CD	91% had previous exposure to biologics	Patients ≥ 40 kg were randomized to 130 mg vs 390 mg or in patients < 40kg: 3 mg/kg vs 9 mg/kg.	At Week 8, patients ≥ 40 kg single SC UST maintenance dose of 9 mg. Patients < 40kg 2 mg/kg of SC UST.
Kakiuchi and Yoshiura^[43]^	Case report	UC	IFX	Intravenous induction, 260 mg of UST	90 mg of SC UST q8w
Yerushalmy-Feler et al^[37]^	Cohort	CD	≈ 99% Biologic experienced	Intravenous induction, 260 mg of UST	90 mg of SC UST q8w then escalated

q6w = every 6 weeks, q8w = every 8 weeks, q12 = every 12 weeks, ADA = adalimumab, IFX = infliximab, CIMZIA = certolizumab, CD = Crohn disease, IBD = inflammatory bowel disease, IBDU = IBD-unclassified, GOL = golimumab, SC = subcutaneous, UC = ulcerative coltis, UST = Ustekinumab, VDZ = vedolizumab.

## Author contributions

**Conceptualization:** Marouf Alhalabi.

**Data curation:** Marouf Alhalabi.

**Methodology:** Marouf Alhalabi.

**Supervision:** Marouf Alhalabi.

**Validation:** Marouf Alhalabi.

**Visualization:** Marouf Alhalabi.

**Writing – original draft:** Marouf Alhalabi.

**Writing – review & editing:** Marouf Alhalabi.
